# Myosin 10 is involved in murine pigmentation

**DOI:** 10.1111/exd.13528

**Published:** 2018-04-24

**Authors:** Kifayathullah Liakath‐Ali, Valerie E. Vancollie, Inês Sequeira, Christopher J. Lelliott, Fiona M. Watt

**Affiliations:** ^1^ Centre for Stem Cells & Regenerative Medicine King's College London London UK; ^2^ Wellcome Trust Sanger Institute, Genome Campus Cambridge UK

**Keywords:** hair follicles, melanocytes, myosin, pigmentation

## Abstract

Myosins are molecular motors that are well known for their role in cell movement and contractile functions. Although extensively studied in muscle physiology, little is known about the function of myosins in mammalian skin. As part of the Sanger Institute Mouse Genetics Project, we have identified a role for *Myo10* in pigmentation, with a phenotype unlike those of *Myo5a* or *Myo7a*. Adult mice homozygous for a disrupted *Myo10* allele on a C57BL/6N background displayed a high degree of penetrance for white patches on their abdomen and dorsal surface. Forepaw syndactyly and hind paw syndactyly were also observed in these mice. Tail epidermal wholemounts showed a complete lack of melanocytes in the hair follicles and interfollicular epidermis. *Myo10* has previously been implicated in human pigmentation. Our current study reveals involvement of *Myo10* in murine skin pigmentation.

**Abbreviations**
ATP: adenosine triphosphate
Hair‐GEL: Hair Gene Expression Library
*Myo10*: Myosin 10
*Myo5a*: Myosin 5a
*Myo7a*: Myosin 7a
Trp1: tyrosinase‐related protein 1
UV: ultraviolet
WT: wild type

## 1 BACKGROUND

Skin pigmentation is a highly variable and conserved trait among living organisms. It plays a critical role in social communication, camouflage, mimicry and protection against the harmful effect of UV radiation.^1^ Melanin pigments mediate the pigmentation process in skin. Melanogenesis is a process whereby melanin pigments are produced and deposited in melanocytes under complex regulatory control by multiple cellular events. Upon maturation, melanosomes—the melanin‐containing granules—are transported from melanocytes to neighbouring keratinocytes in the epidermis.^2^, ^3^

Myosins are among the motor proteins involved in melanosome cargo transportation. Functionally, myosins are a large superfamily of ATP‐dependent motor proteins that translocate actin filaments. Other functions include cell adhesion, motility, endocytosis, exocytosis and cytokinesis.^4^ There are 35 classes of myosins that have been classified based on evolutionary conservation of their motor domains.^5^, ^6^ Myosin 5a (*Myo5a*), Myosin 7a (*Myo7a*) and Myosin 10 (*Myo10*) are unconventional myosins and are known to have functions in melanocytes and neurons.^7^, ^8^ The roles of *Myo5a* and *Myo7a* are known in skin pigmentation, whereas the in vivo involvement of *Myo10*, while previously studied in primary human cell cultures^9^ and being advertised as a key target by at least one commercial cosmetic product, is not fully understood.^10^

## 2 QUESTION ADDRESSED

Does Myosin 10 play a role in murine skin pigmentation?

## 3 EXPERIMENTAL DESIGN

**Figure 1:**
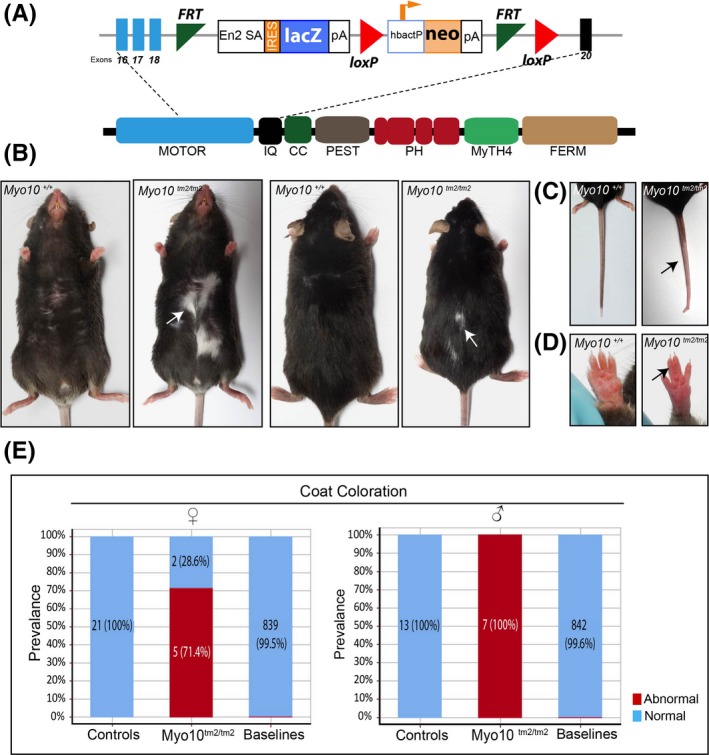
*Myo10* disruption leads to digit and pigmentation defects. (A) Schematic diagram of the deletion (tm2) construct for *Myo10*. Exon numbers indicated (with exon 19 deleted) and corresponding protein domains are illustrated. (B) Coat of adult C57BL/6N *Myo10* ^*tm2/tm2*^ mice. (C) Pale tail phenotype in *Myo10* ^*tm2/tm2*^ mice (assessed in 3 *Myo10* ^*tm2/tm2*^ mice). (D) Fused digits in forepaws of *Myo10* ^*tm2/tm2*^ mice. Digit fusion prevalence: Forepaw: 10/14 *Myo10* ^*tm2/tm2*^ abnormal vs 0/34 local WTs and 0/1688 baseline WTs. Hind paw: 8/14 *Myo10* ^*tm2/tm2*^ vs 0/34 local WTs and 0/1688 baseline WTs. (E) Stacked bar graphs show coat colour abnormalities. Legend—Control, WT mice phenotyped each week alongside each batch of *Myo10* ^*tm2/tm2*^ mice to ensure standard testing conditions; Baseline, cumulative C57BL/6N controls over lifetime of phenotyping pipeline

To investigate the role of Myo10 in mouse skin pigmentation, we analysed *Myo10* ^*tm2/tm2*^ C57BL/6N mice from the Sanger Institute Mouse Genetics Project,^11^ which carry an inserted cassette 3′ to exon 18 and an in‐frame deletion of exon 19 in *Myo10* (Figure 1A). See Data S1.

## 4 RESULTS

To assess the efficiency of knockout of the *Myo10* allele, we performed real time‐qPCR analysis, which showed that *Myo10* ^*tm2/tm2*^ mice have ~15% of wild‐type *Myo10* transcript levels (Figure S1A). Similar to the reported *Myo10* ^*m1J*^ allele^10^ (http://www.informatics.jax.org/allele/MGI:5578506), *Myo10* ^*tm2/tm2*^ mice typically displayed abnormal dorsoventral coat patterning with white belly spots and extensive depigmented areas in the tail. Depigmentation was limited to the tail tip in control mice (Figure 1B‐C). *Myo10* ^*tm2/tm2*^ mice showed ocular abnormalities and also typically presented with interdigital webbing—a condition in which the skin between paw digits is not lost during development (Figure 1D). X‐ray analysis confirmed that this abnormality is not due to fusion of bones (http://www.mousephenotype.org/data/genes/MGI:107716). Unlike Xenopus *Myo10* mutants, *Myo10* ^*tm2/tm2*^ mice did not show any cranial or skeletal abnormalities.^12^ Abnormal coat colour pattern was observed in all male mutants, whereas females showed slightly reduced penetrance of the phenotype (Figure 1E). Skin histopathology revealed no obvious structural abnormalities in the epidermis or dermis (Figure S1B).

**Figure 2:**
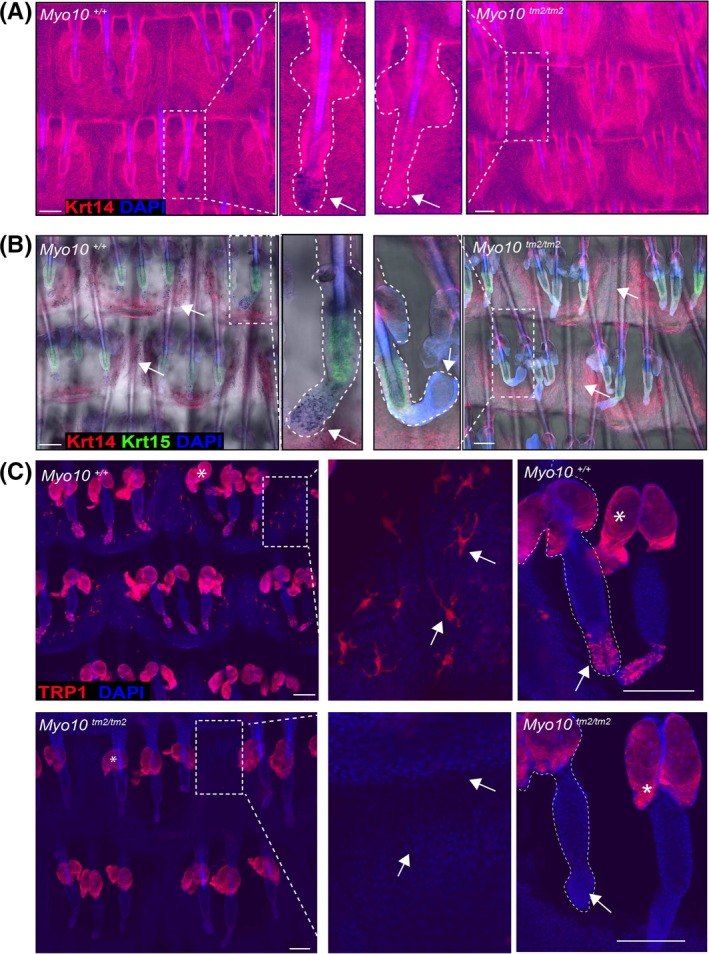
Loss of pigmentation markers in *Myo10* ^*tm2/tm2*^ epidermis and hair follicles. (A) 3D maximal projected epidermal wholemount images show absence of melanocytes in hair follicles in mutant mice when compared to WT (arrows). (B) Bright field images combined with fluorescent labelling confirm the lack of melanocytes in hair follicles (arrows). (C) Single Z‐plane image of immunolabelled epidermal wholemounts of mutant mice with antibody to the melanocyte differentiation marker Trp1 shows the absence of melanocytes in interfollicular epidermis and hair follicles when compared to WT (arrows). Asterisks indicate nonspecific staining in sebaceous glands. Krt14, keratin 14; Krt15, keratin 15; Trp1, tyrosinase‐related protein 1; DAPI, 4′,6‐diamidino‐2‐phenylindole; Scale bars 100 μm

The apparent lack of pigment in the tail of *Myo10* ^*tm2/tm2*^ mice prompted us to investigate whether the skin had any melanocyte abnormalities. Confocal imaging of tail epidermal wholemounts revealed a complete lack of pigmented melanocytes in the hair follicles and interfollicular epidermis (Figure 2A). Similar results were observed by imaging the wholemounts under bright field combined with fluorescent detectors (Figure 2B). We confirmed the lack of melanocyte stem cells and differentiated melanocytes by labelling with antibodies to c‐Kit and tyrosinase‐related protein 1 (Trp1), respectively. Immunostaining for c‐Kit and Trp1 showed no staining in mutant hair follicles and interfollicular epidermis, whereas distinct accumulation of melanocytes with characteristic dendritic projections was observed in WT epidermis (Figure 2C; Figure S1C). This indicates that melanocyte distribution in the epidermis requires *Myo10*.

As Myo5a, Myo7a and Myo10 are unconventional myosins and loss of function mutations result in different patterns of pigmentation defects, we explored putative links in predicted protein‐protein interaction networks. STRING network analysis (http://www.string-db.org/) showed two distinct clusters of interactions for Myo5a and Myo10 (Figure S1D). Myo5a is predicted to interact with Myo7a. The other interaction partners are mostly exocyst complex component (Exoc) genes, which are involved in the docking of exocytic vesicles.^13^

Surprisingly, the interaction network of Myo10 was separate from that of Myo5a. Top scoring predicted interaction partners included Itgb5, Calm13 and Neo1, all of which are expressed in the plasma membrane. The distinct class of interactions of Myo10 and Myo5a suggests that Myo10 may play a different role from Myo5a in pigmentation. *Myo10* is highly expressed in mouse skin,^14^ so we analysed expression of *Myo10* in different skin compartments using a publicly available skin gene expression (Hair‐GEL) database.^15^ Interestingly, while *Myo10* is predominantly expressed in melanocytes in embryonic and neonatal mice, it is also expressed in other skin compartments such as epidermis and dermal fibroblasts (Figure S1E).

## 5 CONCLUSIONS

The role of myosins in melanoblast migration is known through studies on Rac1 mouse mutants,^16^, ^17^ although direct evidence implicating the involvement of Myo10 has not been reported. An actin‐bundling protein, Fascin1 (Fscn1), which is a key interacting partner of Myo10, is known to promote migration and proliferation of melanoblasts.^18^ In the network analysis of Myo10 interaction partners, we found Fscn1 as a key protein that is known to physically interact with Myo10. This suggests that Myo10 may have a similar role to Fscn1 in actin bundling.

Like most cells, melanoblasts migrate with the help of cytoplasmic projections called filopodia.^19^, ^20^ HeLa cells that lack *Myo10* fail to form filopodia,^21^ indicating that *Myo10* is an essential component for filopodia formation. In addition to its role in melanoblast migration, *Myo10* has been shown to function in melanosome transportation. Exposure to UV light increases the number of filopodia and *Myo10* expression in differentiated melanocytes. Knockdown of *Myo10* leads to decreased numbers of filopodia and reduced melanosome transfer.^9^ The digit webbing phenotype may also indicate a role for Myo10 in apoptosis, for example as a dependence receptor required for apoptosis induction. Recent data suggest that Myo10 determines tumour invasiveness and cell cycle regulation (reviewed in),^22^ leading us to suggest that it could exert these functions both in melanocytes and in other skin cell populations.

While *Myo10* mutant tail epidermis lacked melanocytes, dorsal and ventral skin had a chimeric pattern of normal and abnormal pigmentation. It is intriguing that the pigmentation process in the tail is distinct from dorsoventral skin and reduced penetrance of the phenotype among females, although the exact mechanism of this for sexual dimorphism is unclear.^23^ Developmental profiling of melanoblast migration using *Myo10* mutants during embryogenesis would be helpful to address this phenomenon. It is also possible that *Myo10* has other cellular roles in the skin, for example during wound healing or tumour formation.

## CONFLICT OF INTEREST

The authors have declared no conflicting interest.

## AUTHOR CONTRIBUTIONS

KL, CJL and FMW designed the study. KL, VEV and IS performed the experiments. KL, VEV, IS, CJL and FMW analysed and interpreted the data. KL and CJL wrote the manuscript with input from all authors. All authors approved the submitted manuscript.

## Supporting information


**Figure S1** Histology of *Myo10* ^*tm2/tm2*^ skin, interaction network comparison of *Myo10* and *Myo5a* and expression of *Myo10*. (A) *Myo10* expression in WT and *Myo10* ^*tm2/tm2*^ tail. *Myo10* probe spans exons 28‐29. RQ value is relative to endogenous control gene B2m. (B) Haematoxylin and eosin staining of the tail and dorsal skin of *Myo10* ^*tm2/tm2*^ and WT mice. (C) *Myo10* ^*tm2/tm2*^ tail skin sections immunolabelled with the antibody to the melanocyte stem cell marker c‐Kit show the absence of melanocytes in hair follicles when compared to WT (arrows). Asterisks indicate nonspecific staining. (D) STRING interaction network of Myo10 and Myo5a shows two distinct classes of interacting proteins. (E) Gene expression results obtained from Hair‐GEL database show high expression of *Myo10* in melanocytes at E14.5 and widespread expression in all skin subpopulations at P5. FPKM, Fragments Per Kilobase of transcript per Million mapped reads; Scale bars 100 μm.

**Data S1** Materials and Methods
