# Supplementary figures and images for: Myosin 10 is involved in murine pigmentation
Source: Exp Dermatol. 2018 Apr 24;28(4):391–4. doi: 10.1111/exd.13528 (PMC6519374; doi:10.1111/exd.13528)

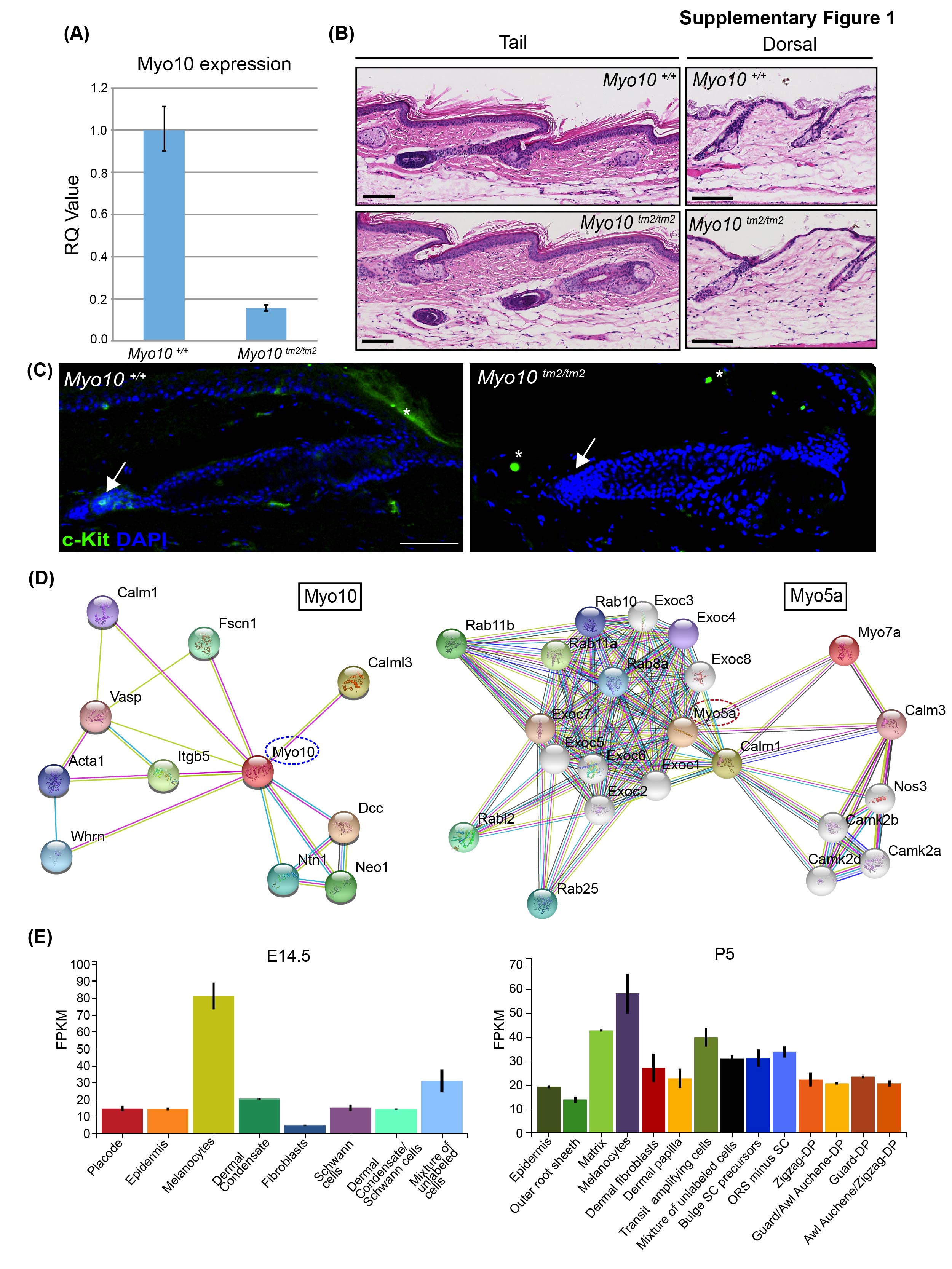

Supplement: Supplementary file 1 — Figure S1 Histology of Myo10 tm2/tm2 skin, interaction network comparison of Myo10 and Myo5a and expression of Myo10. (A) Myo10 expression in WT and Myo10 tm2/tm2 tail. Myo10 probe spans exons 28‐29. RQ value is relative to endogenous control gene B2m. (B) Haematoxylin and eosin staining of the tail and dorsal skin of Myo10 tm2/tm2 and WT mice. (C) Myo10 tm2/tm2 tail skin sections immunolabelled with the antibody to the melanocyte stem cell marker c‐Kit show the absence of melanocytes in hair follicles when compared to WT (arrows). Asterisks indicate nonspecific staining. (D) STRING interaction network of Myo10 and Myo5a shows two distinct classes of interacting proteins. (E) Gene expression results obtained from Hair‐GEL database show high expression of Myo10 in melanocytes at E14.5 and widespread expression in all skin subpopulations at P5. FPKM, Fragments Per Kilobase of transcript per Million mapped reads; Scale bars 100 μm. [file EXD-28-391-s001.jpg]
