# Supplementary material for: Myosin 10 is involved in murine pigmentation
Source: Exp Dermatol. 2018 Apr 24;28(4):391–4. doi: 10.1111/exd.13528 (PMC6519374; doi:10.1111/exd.13528)
Supplement: Supplementary file 2 — Data S1 Materials and Methods [file EXD-28-391-s002.docx]

**Materials and Methods**

**Knockout mice generation and husbandry**

The care and use of all animals were carried out in accordance with UK Home Office regulations, UK Animals (Scientific Procedures) Act 1986 and with approval from Wellcome Trust Sanger Institute’s Animal Welfare and Ethical Review Body. The *Myo10* knockout mouse line (*Myo10^tm2(KOMP)Wtsi^*) was generated as part of the Sanger Mouse Genetics Project. Targeted gene targeting and preliminary phenotyping strategies previously reported elsewhere [s1, s2]. The *Myo10* gene was targeted in C57BL/6N ES cells with a knockout-first conditional-ready cassette (clone EPD0272_4_F10) with genotyping carried out as previously described (s3). Animals were maintained on the C57BL/6N background in a specific pathogen free environment with ad libitum access to food (Mouse Breeders Diet (LabDiets 5021-3, IPS, Richmond, USA) and water. The housing environment were individually ventilated cages at standard temperature (19-23°C) and humidity (55% ±10%), on a 12-h dark, 12-h light cycle (lights on 07:00–19:00 h). Mice were typically housed in cage densities of 3-5 per cage. In addition to Aspen bedding substrate, standard environmental enrichment of two nestlets, a cardboard tunnel and three wooden chew blocks were provided.

**Expression analysis of *Myo10***

For qPCR analysis, RNA from tail was extracted from 16 week-old mice and subjected to RT–qPCR using an RNA-to-Ct One-Step Kit (Life Technologies, Carlsbad, CA, USA), using TaqMan assay for mouse *Myo10* (Mm00450859_m1 FAM-MGB, spanning exons 28-29) and a *B2m* primer-limited endogenous control (Mm00437762_m1 VIC-MGB_PL; all from Life Technologies) to normalize for variations between the amounts of input RNA. Reactions were performed in triplicate using a ViiA 7 qPCR machine (Life Technologies) and analysed using the 2–ΔΔCT method.

**Phenotyping**

Mice undergoing primary phenotyping, including protocols for an initial dysmorphology assessment at 9 weeks and x-ray assessment at 13 weeks, were studied using a modified version of the Sanger Mouse Genetics Project, detailed previously [s2], using Mouse Breeders Diet instead of a high-fat diet and without 4 previously run tests (hair phenotyping, open field, hot plate and stress induced hypothermia tests). Based on the primary screen data, whole-body morphology, including examination of the hair, skin and vibrissae, was performed using a standardized checklist of 104 parameters and, where appropriate, images were captured at various ages using a Sony DSC-HX7V. Further information about these parameters can be found at [https://www.mousephenotype.org/impress/protocol/185/15](https://www.mousephenotype.org/impress/protocol/185/15).

Due to the high-throughput nature of the pipeline, with mice being processed with different mutant alleles during a single session, for mouse management purposes, the cages have both genotype and allele information displayed. However, multiple small batches of mutant mice are phenotyped over a period to time to minimize cage, batch and investigator effects, alongside weekly wild type controls (7 males and 7 females). For homozygous *Myo10^tm2(KOMP)Wtsi^* mutants, 5 batches were analyzed (3 males, 3 males and 3 females, 4 males, 3 females, 1 female) over a 5 month period.

Additional mouse phenotyping data and images from the primary pipeline can be found at the International Mouse Phenotyping Consortium website (<http://www.mousephenotype.org/data/genes/MGI:107716>)

**Phenotyping Statistics**

Primary dysmorphological observations at 9 weeks were analysed compared to a global reference range combining all wild-type mice run in the pipeline, as described in [s2], with overall prevalences stated in the figures. WTSI phenotypes mice from multiple mutant colonies in a single week, and for a given colony, we run multiple small batches of mutants over several weeks (a so-called multibatch strategy). In order to be able to detect phenotypes, we have two different control strategies. Firstly, we always run age, sex and strain matched wild types every week alongside our mutant mice. In the graph, the control group labelled “Controls” are the accumulated weekly controls that were phenotyped alongside any batches of Myo10 mutants. These local WT controls act as quality controls for various tests in the screen, for example ensure that machines were properly calibrated, or to demonstrate that background characteristics of the strain is not changing.

In addition, we aggregate the data from all age, sex and strain matched wild types irrespective of whether they were run alongside Myo10 mutant mice. This is the control data labelled “Baseline”, and shows the prevalence and range of a given phenotype in the population, akin to using clinical reference ranges when examining blood values from a patient. Evaluating the graphs in Figure 1e, none of the local controls run alongside the *Myo10^tm2/tm2^*  display abnormalities in coat coloration. In addition, from nearly 1700 examined age, sex and strain matched wild types, less than 1% showed an abnormal coat coloration. This demonstrates that the phenotype seen in the *Myo10^tm2/tm2^*  are very rarely seen in the wild type background and is highly likely to be a true phenotype in the *Myo10^tm2/tm2^* mutants.

**Epidermal wholemounts and imaging**

The epidermal wholemount immuno-labelling procedure was performed as described previously [s4, s5]. In brief, mouse tail was slit on the ventral side lengthways. Skin pieces (0.5x0.5cm2) were incubated in 5mM EDTA in PBS for 4h at 37°C. Epidermis was gently peeled from dermis as an intact sheet in a proximal to distal direction, corresponding to the orientation of the hairs, and then the epidermis was fixed in 4% paraformaldehyde (PFA; Sigma) for 1h at room temperature. Fixed epidermal sheets were washed in PBS and stored in PBS containing 0.2% sodium azide at 4°C. Confocal image acquisition of stained wholemounts and skin sections were performed using a Nikon A1 confocal microscope. Images were analysed using NIS Elements (Nikon Instruments Inc.).

**Histology**

Skin samples were fixed with 10% neutral buffered formalin overnight before paraffin embedding. The tissues were sectioned and stained with haematoxylin and eosin (H&E) by conventional methods.

**Antibodies**

Primary antibodies for epidermal wholemount were: directly conjugated (AlexaFluor 555) Krt14 (LL002, in house, 1:200); directly conjugated (AlexaFluor 488) Krt15 (LHK-15, in-house, 1:50); mouse anti-TRP1 (clone TA99, Abcam ab3312, 1:200) and rat ant-c-Kit (CD117, BD pharmigen, clone ACK45, cat 553868, 1:100).

**Network and gene expression analysis**

Direct (physical) and indirect (functional) protein associations were obtained from the STRING database [s6]. Skin and hair follicle subpopulation gene expression was obtained from Hair-GEL database [s7, s8].

**Supplemental References**

**s1**. Skarnes W C, Rosen B, West A P , Koutsourakis M, Bushell W, Iyer V, Mujica AO, Thomas M, Harrow J, Cox T, Jackson D, Severin J, Biggs P, Fu J, Nefedov M, de Jong PJ, Stewart AF, Bradley A. A conditional knockout resource for the genome-wide study of mouse gene function. Nature 2011: 474: 337-342.

**s2**. White J K, Gerdin A K, Karp N A, Ryder E, Buljan M, Bussell JN, Salisbury J, Clare S, Ingham NJ, Podrini C, Houghton R, Estabel J, Bottomley JR, Melvin DG, Sunter D, Adams NC; Sanger Institute Mouse Genetics Project, Tannahill D, Logan DW, Macarthur DG, Flint J, Mahajan VB, Tsang SH, Smyth I, Watt FM, Skarnes WC, Dougan G, Adams DJ, Ramirez-Solis R, Bradley A, Steel KP.. Genome-wide generation and systematic phenotyping of knockout mice reveals new roles for many genes. Cell 2013: 154: 452-464.

**s3**. Ryder E, Gleeson D, Sethi D, Vyas S, Miklejewska E, Dalvi P, Habib B, Cook R, Hardy M, Jhaveri K, Bottomley J, Wardle-Jones H, Bussell JN, Houghton R, Salisbury J, Skarnes WC; Sanger Mouse Genetics Project, Ramirez-Solis R. Molecular characterization of mutant mouse strains generated from the EUCOMM/KOMP-CSD ES cell resource. Mammalian genome: official journal of the International Mammalian Genome Society 2013: 24: 286-294.

**s4**. Braun K M, Niemann C, Jensen U B, Sundberg JP, Silva-Vargas V, Watt FM. Manipulation of stem cell proliferation and lineage commitment: visualisation of label-retaining cells in wholemounts of mouse epidermis. Development 2003: 130: 5241-5255.

**S5**. Liakath-Ali K, Vancollie VE, Heath E, Smedley DP, Estabel J, Sunter D, Ditommaso T, White JK, Ramirez-Solis R, Smyth I, Steel KP, Watt FM. Novel skin phenotypes revealed by a genome-wide mouse reverse genetic screen. Nat Commun 2014: 5: 3540.

**s7**. Franceschini A, Szklarczyk D, Frankild S, Kuhn M, Simonovic M, Roth A, Lin J, Minguez P, Bork P, von Mering C, Jensen LJ. STRING v9.1: protein-protein interaction networks, with increased coverage and integration. Nucleic Acids Res 2013: 41: D808-815.

**s8**. Rezza A, Wang Z, Sennett R, Qiao W, Wang D, Heitman N, Mok KW, Clavel C, Yi R, Zandstra P, Ma'ayan A, Rendl M. Signaling Networks among Stem Cell Precursors, Transit-Amplifying Progenitors, and their Niche in Developing Hair Follicles. Cell Rep 2016: 14: 3001-3018.

**s9**. Sennett R, Wang Z, Rezza A, Grisanti L, Roitershtein N, Sicchio C, Mok KW, Heitman NJ, Clavel C, Ma'ayan A, Rendl M. An Integrated Transcriptome Atlas of Embryonic Hair Follicle Progenitors, Their Niche, and the Developing Skin. Dev Cell 2015: 34: 577-591.
